# Regulation of Osteoimmune Microenvironment and Osteogenesis by 3D‐Printed PLAG/black Phosphorus Scaffolds for Bone Regeneration

**DOI:** 10.1002/advs.202302539

**Published:** 2023-08-24

**Authors:** Jing Long, Zhenyu Yao, Wei Zhang, Ben Liu, Kaiming Chen, Long Li, Bin Teng, Xiang‐Fu Du, Cairong Li, Xue‐Feng Yu, Ling Qin, Yuxiao Lai

**Affiliations:** ^1^ Centre for Translational Medicine Research & Development Shenzhen Institute of Advanced Technology Chinese Academy of Sciences Shenzhen 518055 P. R. China; ^2^ Center for Energy Metabolism and Reproduction Shenzhen Institute of Advanced Technology Chinese Academy of Sciences Shenzhen 518055 P. R. China; ^3^ Materials and Interfaces Center Shenzhen Institute of Advanced Technology Chinese Academy of Sciences Shenzhen 518055 P. R. China; ^4^ Musculoskeletal Research Laboratory Department of Orthopaedics & Traumatology The Chinese University of Hong Kong HK Hong Kong SAR 999077 P. R. China; ^5^ CAS‐HK Joint Lab of Biomaterials Shenzhen 518055 P. R. China; ^6^ Guangdong Engineering Laboratory of Biomaterials Additive Manufacturing Shenzhen 518055 P. R. China; ^7^ Orthopaedics/Department of Spine Surgery the First Affiliated Hospital, Shenzhen University, Shenzhen Second People’s Hospital Shenzhen 518035 P. R. China

**Keywords:** 3D‐printed Scaffolds, black phosphorus, bone regeneration, osteoimmune microenvironment, macrophage polarization

## Abstract

The treatment of bone defects remains a significant challenge to be solved clinically. Immunomodulatory properties of orthopedic biomaterials have significance in regulating osteoimmune microenvironment for osteogenesis. A lactic acid‐co‐glycolic acid (PLGA) scaffold incorporates black phosphorus (BP) fabricated by 3D printing technology to investigate the effect of BP on osteoimmunomodulation and osteogenesis in site. The PLGA/BP scaffold exhibits suitable biocompatibility, biodegradability, and mechanical properties as an excellent microenvironment to support new bone formation. The studies' result also demonstrate that the PLGA/BP scaffolds are able to recruit and stimulate macrophages M2 polarization, inhibit inflammation, and promote human bone marrow mesenchymal stem cells (hBMSCs) proliferation and differentiation, which in turn promotes bone regeneration in the distal femoral defect region of steroid‐associated osteonecrosis (SAON) rat model. Moreover, it is screened and demonstrated that PLGA/BP scaffolds can promote osteogenic differentiation by transcriptomic analysis, and PLGA/BP scaffolds promote osteogenic differentiation and mineralization by activating PI3K‐AKT signaling pathway in hBMSC cells. In this study, it is shown that the innovative PLGA/BP scaffolds are extremely effective in stimulating bone regeneration by regulating macrophage M2 polarization and a new strategy for the development of biomaterials that can be used to repair bone defects is offered.

## Graphical abstract

A PLGA/BP scaffold fabricated by 3D printing technology to improve the bone immune microenvironment and promote bone regeneration is intoduced. PLGA/BP scaffolds modulate the polarization of macrophages from M1 to M2, effectively inhibiting the inflammatory response. Furthermore, the activation of the PI3K‐AKT pathways by PLGA/BP scaffolds promotes the transcriptional regulation of Runx2 and IBSP, SPP1, therefore promoting osteogenesis and bone regeneration.

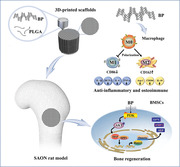

## 1 Introduction

Bone defects caused by severe trauma, bone tumor, or osteomyelitis remain common in clinical orthopedics.^[^ ^1^ ^]^ The issue of bone regeneration still remains a major challenge in clinical surgery, despite many therapeutic options like autografts, allografts, and artificial scaffolds.^[^ ^2^ ^]^ With the development of biomaterials in recent years, biomaterials in the form of 3D‐printed scaffolds have been extensively utilized in bone regeneration research.^[^ ^3^ ^]^ Due to its personalized design and good biocompatibility, it can accelerate bone regeneration by improving surgical accuracy and safety.^[^ ^4^ ^]^ Despite this, there are some problems associated with the implantation of 3D‐printed scaffolds in patients. When scaffolds are implanted into bone defects, immune cells are actively migrated around the biomaterials. After implantation, biomaterials initially undergo an inflammatory phase, and the behavior of the inflammatory phase plays a crucial role in subsequent tissue regeneration. A moderate inflammatory response causes macrophages to express an anti‐inflammatory and pro‐regeneration phenotype, secrete a large number of cytokines, and activate somatic cells, which promotes wound healing.^[^ ^5^ ^]^ Similar to the natural repair process after tissue injury, the interactive reaction between biomaterials and the immune system cannot be avoided when the regenerative system is activated by biomaterials.^[^ ^6^ ^]^ Therefore, the development of bone substitute biomaterials should promote osteogenic differentiation and focus on the induction of a moderate osteoimmune microenvironment.

Macrophages play a very important role in regulating the inflammatory response and tissue regeneration.^[^ ^7^ ^]^ Essentially, monocytes recruit to the implanted biomaterials surface and polarize into M1 or M2‐type macrophages. M1 macrophages produce pro‐inflammatory cytokines like tumor necrosis factor‐α (TNF‐α), interleukin‐6 (IL‐6), monocyte chemotactic protein‐1 (MCP‐1), and interleukin‐1β (IL‐1β) in the bone tissue microenvironment, whereas M2 macrophages produce anti‐inflammatory cytokines such as Arginase 1（Arg‐1）, interleukin‐4 (IL‐4), interleukin‐10 (IL‐10), interleukin‐13 (IL‐13), and cluster of differentiation 206 (CD206), which inhibit inflammation and promote osteogenesis and angiogenesis.^[^ ^6^, ^8^ ^]^ Through M1/M2 phenotype switching and secretion of various cytokines, fibroblasts, BMSCs, and osteoprogenitor cells are attracted to biomaterials, undergo the initial inflammatory phase, and create a favorable osteoimmune microenvironment for subsequent bone healing.^[^ ^9^ ^]^

BP is a novel 2D material that researchers have paid more attention to in recent years due to its unique and excellent properties.^[^ ^10^ ^]^ BP has a layer‐dependent band gap and exhibits a high surface‐to‐volume ratio, excellent biocompatibility, and biodegradability.^[^ ^10^ ^]^ These properties offer unlimited potential for biological applications such as photodynamic therapy (PTT), photothermal therapy (PDT), and drug delivery.^[^ ^11^ ^]^ Compared to other 2D materials, BP is biocompatible and biodegradable in the physiological environment. Phosphate anions are nontoxic degradation products of BP, which are important for bone tissue mineralization. While it has been reported that biomaterials based on BP can promote bone regeneration,^[^ ^12^ ^]^ little is known about whether BP regulates the immunological microenvironment of bone.

SAON is a common orthopedic disease caused by the long‐term and massive use of corticosteroid therapy attributed to its effects to treat some immune diseases or save lives in clinical practice. Core decompression is an important measure for early intervention and treatment of steroid‐induced osteonecrosis, which can reduce the intraosseous pressure in the necrotic area and restore blood supply to the necrotic area, which promotes bone repair.^[^ ^13^ ^]^ Therefore, we further researched the immunomodulatory functions using the SAON rat model, since lipopolysaccharide (LPS) and a high dose of methylprednisolone MPS were injected initially, the early injection of LPS can induce an inflammatory response, so we used this model to study the immunomodulatory function of BP.^[^ ^14^ ^]^

**Scheme 1:**
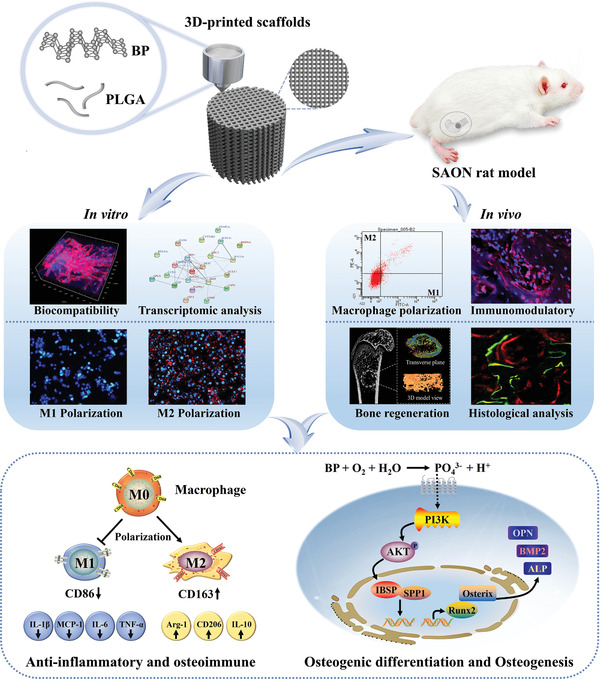
Schematic illustration of PLGA/BP scaffolds fabricated by 3D printing and proposed mechanism of osteoimmune environment induced by BP degradation to accelerate bone regeneration.

The PLGA has been approved by the United States Food and Drug Administration (FDA) for its good biocompatibility and biodegradability. The encapsulation effect of PLGA can slow release the degradation of BP nanosheets, which is conducive to 3D printing technology to accurately regulate the porosity, pore size, pore distribution, pore connectivity, and other properties of porous scaffolds for bone repair materials. To investigate the function of BP on osteogenesis in bone regeneration and the osteoimmunomodulatory effect in the osteoimmune microenvironment, we developed PLGA incorporated BP nanosheets scaffolds using low‐temperature 3D printing technology (PLGA/BP scaffolds) to meet the special requirements of various osteogenic material structure and the biological and mechanical requirements. We investigated the osteoimmunomodulatory effect of PLGA/BP scaffolds on converted macrophages from the M1 to the M2 phenotype in vitro. Finally, PLGA/BP scaffolds were implanted into a SAON rat model to investigate osteoimmunomodulatory effects and bone regeneration. This study provides evidence that PLGA/BP scaffolds have osteogenic effects and osteoimmunomodulatory functions conducive to bone regeneration (**Scheme** **1**).

## 2 Results and Discussion

### 2.1 Fabrication and Characterization of 3D‐Printed BP Nanosheets as Scaffolds

**Figure 1:**
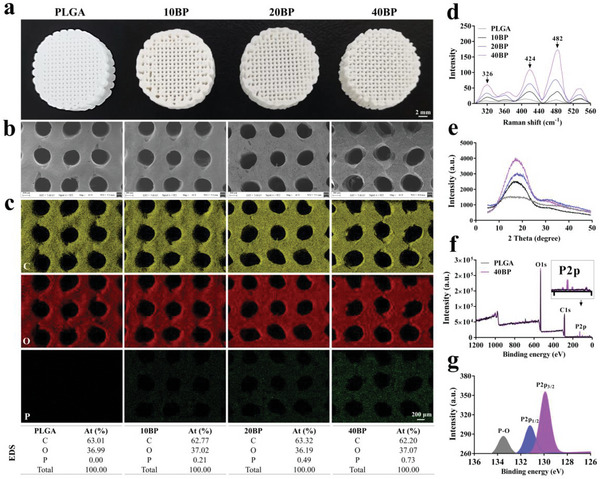
Morphology and characterizations of 3D printing scaffolds. a) Digital photos of the PLGA/BP scaffolds with different compositions; BP nanosheets of the total content were 0 wt.% (PLGA), 0.01 wt.% (10BP), 0.02 wt.% (20BP), and 0.04 wt.% (40BP), respectively. b) Morphology observation of different compositions scaffold by SEM. c) Energy Dispersive Spectrometer (SEM‐EDS) of the transverse section of different scaffolds. Element carbon (C, yellow); Element Oxygen (O, red); Element phosphorus (P, green). d) Raman spectrum of PLGA/BP scaffolds with different contents. e) XRD patterns of PLGA/BP scaffolds with different contents. f) XPS spectra of the PLGA and 40BP scaffolds. g) XPS spectra of P2p3/2, P2p1/2, and P‐O for 40BP scaffolds. *n* = 3 independent samples.

In our study, low‐temperature deposition 3D printing technology was utilized to fabricate PLGA/BP scaffolds. The biodegradable polymer PLGA was used as a matrix for the formation of composite scaffolds. The FDA has approved PLGA as one of the most used biomedical polymers.^[^ ^15^ ^]^ In biomedicine, BP nanomaterials possess several advantages, including optical properties, excellent biocompatibility, and biodegradability. For the following studies, the scaffolds were designed and synthesized as follows. Different amounts of BP nanosheets were added to a 1,4‐dioxane solution of 15 wt.% PLGA with different BP nanosheets contents of 0 wt.% (PLGA), 0.01 wt.% (10BP), 0.02 wt.% (20BP), and 0.04 wt.% (40BP), respectively, and fabricated by a low‐temperature 3D printing technology at −30 °C (**Figure** **1**a). By using low‐temperature 3D printing technology to modulate the porosity of scaffolds, the PLGA/BP scaffolds had high interconnectivity characterized with macropores and micropores, which could increase osteoconductivity, promote the proliferation of bone cells and their adhesion to the extracellular matrix. Scanning electron micrographs (SEM) of PLGA/BP scaffolds with different BP concentrations demonstrated that the scaffolds had a well‐interconnected structure with an average macropore lateral size of 408.69 ± 29.76 µm, and micropores on the scaffolds surface with a lateral size of 1–50 µm (Figure 1b; Figure S1c, Supporting Information). SEM combined with energy‐dispersive X‐ray spectroscopy (SEM‐EDS) was used to map the elements in the cross sections of the PLGA/BP scaffolds. The elements oxygen (O, red) and carbon (C, yellow) were found to be uniformly distributed in the frameworks (Figure 1c), while the proportion of phosphorus elements (P, green) in the frameworks increased with increasing BP content. The smooth surface, uniform morphology, and clear edge of the exfoliated BP nanosheets on the silicon substrate (Figure S1a, Supporting Information) show that BP has an excellent crystal structure, transparent and undistorted lamellar structure, confirming thestable structure of the BP nanosheets. Also, the high‐resolution transmission electron microscopy (TEM) image of BP nanosheets showed lattice fringes with a D‐spacing of 0.31 nm (Figure S1b, Supporting Information), consistent with reports of the single‐layer BP structure.^[^ ^16^ ^]^ In addition, the Raman spectrum also showed the structural changes of the BP nanosheets in the PLGA/BP scaffolds with different contents (Figure 1d). In compared with the out‐of‐plane phonon mode (A_g_^1^) and two in‐plane modes (B_2g_ and A_g_^2^) of BP, there are three representative characteristic Raman peaks of PLGA/BP scaffolds at 326, 424, and 482 cm^−1^, respectively.^[^ ^17^ ^]^ These characteristic peaks of the PLAG/BP scaffold with different contents show significant red shifts compared to those of the BP nanosheets reported in the literature, which generally indicates the reduction in the number of layers of the tested materials. The X‐ray diffraction (XRD) patterns of the 40BP scaffolds show clear characteristic peaks at the 17.07^o^ position, corresponding to the (020) crystal plane of the BP (Figure 1e). In addition, the PLGA and 40BP scaffolds were characterized by X‐ray photoelectron spectroscopy (XPS). As shown in Figure 1f, the spectra of the 40BP scaffolds have three peaks at 533, 286, and 128 eV, corresponding to the O1s, C1s, and P2p peaks, respectively, while the PLGA scaffolds have only O1s and C1s peaks, confirming the introduction of BP nanosheets into the PLGA scaffolds. Furthermore, the high‐resolution peak fitting was applied to the P2p peak for the 40BP scaffolds (Figure 1g). It showed that there are 129.9, 131.2, and 133.5 eV peaks corresponding to P2p3/2, P2p1/2, and P‐O, respectively. The percentage of atoms (at%) was 56.93%, 26.04%, and 17.03%, respectively.

### 2.2 In Vitro Studies of PLGA/BP Scaffolds

#### 2.2.1 Degradation and Biocompatibility Properties of PLGA/BP Scaffolds

**Figure 2:**
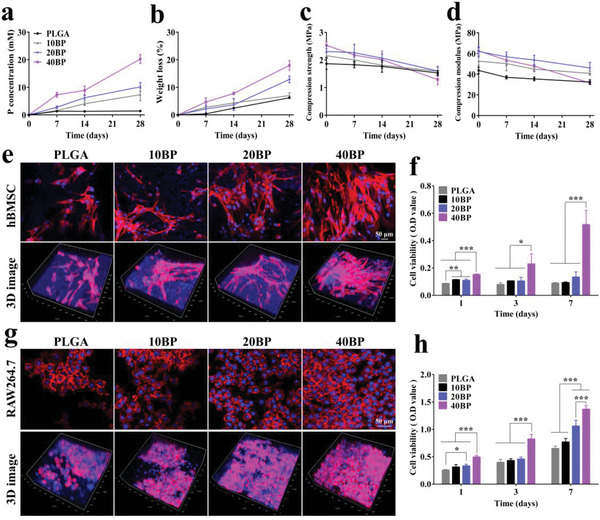
In vitro degradation behavior and biocompatibility tests of PLGA/BP scaffolds with different BP concentrations. The scaffolds were immersed in the PBS solution for 28 days. a–d) The accumulated releasing phosphorus concentration in the PBS solution and the changes in scaffold weight, compressive strength, and compressive modulus after 28 days were determined. e) Confocal laser scanning microscope (CLSM) images of hBMSC cells stained with phalloidin (red) and DAPI (blue) after culturing on the scaffolds after 7 days. f) Relative cell viability of hBMSC cells incubated in the presence of different scaffolds after further cultivation for 1, 3, and 7 days. g) CLSM images of RAW264.7 cells stained with phalloidin (red) and DAPI (blue) after culturing on the BP scaffolds after 7 days. h) Relative cell viability of RAW264.7 cells incubated in the presence of different BP scaffolds after further cultivation for 1, 3, and 7 days. *n* = 3 independent samples, ^*^ *p* < 0.05, ^**^ *p* < 0.01, and ^***^ *p* < 0.001 by one‐way ANOVA with Tukey's post hoc test.

The degradability of PLGA/BP scaffolds was inverstigated in phosphate‐buffered saline (PBS; pH 7.4) at 37 °C. Since the hydrophobic effect of PLGA, the BP nanosheets are adequately protected from oxygen and water, resulting in increased stability of PLGA/BP scaffolds in water under ambient conditions. BP Nanosheets can degrade slowly and release phosphoric acid molecules continuously for 28 days (**Figure** **2**a), and the P element concentration in the PBS also increased continuously, as demonstrated by inductively coupled plasma optical emission spectrometry (ICP‐OES). As BP nanosheets degrade, they are reacted irreversibly with oxygen and water to form phosphorus oxides (P_x_O_y_), which are then converted into the final anions (PO_4_^3−^).^[^ ^18^ ^]^ The weight loss of 40BP scaffolds shows an increasing trend in the first week and accelerates to nearly 20% after 4 weeks (Figure 2b). The final degradation products of the PLGA/BP scaffolds are nontoxic phosphate and lactic acid.^[^ ^19^ ^]^ The changes in compressive strength and compressive modulus of the PLGA/BP scaffolds at indicate degradation time points are shown in Figure 2c,d. Mechanical tests showed that the initial compressive strength of the PLGA/BP scaffolds (2.16 ± 0.09 MPa for 10BP, 2.31 ± 0.21 MPa for 20BP, and 2.53 ± 0.12 MPa for 40BP) was significantly improved than that of the PLGA scaffold (1.87 ± 0.21 MPa), and also the initial compressive modulus of the PLGA/BP scaffolds (52.61 ± 3.96 MPa for 10BP, 62.02 ± 4.22 MPa for 20BP, and 62.57 ± 2.70 MPa for 40BP) was significantly enhanced than that of PLGA scaffolds (43.64 ± 3.07 MPa). From the results, the mechanical properties of the different PLGA/BP scaffolds showed decreasing trends in all groups during the four‐week degradation.

BP nanosheets are characterized by puckered structure and its bilayer zigzag configuration, which gives BP good electronic conductivity, optical, thermoelectric, and other properties.^[^ ^20^ ^]^ It also allows BP nanomaterials to be used in various biomedical applications, such as PDT, PTT, drug delivery, and so on.^[^ ^21^ ^]^ In this studies, combining low‐temperature deposition rapid prototyping 3D‐printing technology with BP nanosheets, PLGA/BP scaffolds exhibit excellent biocompatibility and degradable properties. According to the national standard GB/T 16 886.5‐2017, it is known that for biocompatible materials, the cell viability in their 100% liquid extracts should not be lower than 70% compared to the control, while the PLGA/BP groups in this study were all higher than 100% (Figure S2b, Supporting Information). The BP nanosheets we use are concentrated in the 220 nm range (data not shown), which can effectively cause cell responses and promote osteogenic differentiation. As shown in Figure 2a–d, PLGA/BP scaffolds also exhibit good biodegradability and outstanding mechanical properties. Although it has been reported that BP degrades quickly in the physiological environment,^[^ ^22^ ^]^ which might restrict its application in some aspects,^[^ ^23^ ^]^ in the process of bone regeneration, nontoxic PO_4_^3−^ is the final degradation product of BP, which is a component of bone tissue and a resource for mineralization.^[^ ^22^, ^24^ ^]^ Ca^2+^ can be captured by phosphate, forming calcium phosphate (CaP) deposits that speed up bone repair,^[^ ^25^ ^]^ and phosphorus‐rich materials can stimulate mineralization and accelerate bone regeneration.^[^ ^26^ ^]^ In addition, as shown in Figure 2e–h, we co‐cultured three different concentrations of PLGA/BP scaffolds for 7 days. Subsequently, fluorescence staining of phalloidin was conducted. The experimental results indicate that a significant number of hBMSC or RAW264.7 cells adhering to the scaffold and the abundant cytoskeleton demonstrate the good biocompatibility properties of the material. In summary, our results demonstrated a detailed evaluation of BP as a bone repair biomaterial with multiple properties and revealed that BP can combine with Ca^2+^ to form CaP deposits in the form of PO_4_^3−^, thus accelerating bone formation and promoting bone repair. PLGA/BP scaffolds also have suitable biocompatibility, biodegradability, and outstanding mechanical properties and are ideal for bone defect treatment.

#### 2.2.2 Proliferation and Differentiation of hBMSC Cells on PLGA/BP Scaffolds

The proliferation and differentiation of hBMSC cells on scaffolds were studied by culturing them on the scaffolds for 7 days. Confocal laser scanning microscopy analysis of the staining of the cytoskeleton of hBMSC (Figure 2e) showed that BP nanosheets enhanced cell adhesion and spreading, with strongly elongated actin filaments (red) surrounding the cell nuclei (blue). The cell counting kit (CCK‐8) method measured the cell proliferation activity of hBMSC cells on the PLGA or BP scaffolds (Figure 2f). On day 1, the number of cells adhering to the PLGA/BP scaffolds was significantly increased than in the PLGA group (*p* < 0.01). The number of cells in the 40BP group was the largest and distributed on the internal and external surfaces of the scaffold. The morphology became flat and fusiform, and many filamentous pseudopods extended completely along the wire diameter (Figure 2e). On day 7, the number of cell proliferation in the 40BP group was significantly increased than the PLGA group (*p* < 0.001). The results of cell proliferation of leaching solution of different BP scaffolds confirmed that all different BP concentration scaffolds of degradation release leaching solution could promote hBMSC proliferation to different extents, and 20BP and 40BP groups had more obvious fluorescence intensity distribution by ZEISS ZEN 3.4 software analysis (Figure S2, Supporting Information). These results show that 0.02–0.04 wt.% PLGA/BP scaffolds can significantly promote cell adhesion and proliferation. The PLGA/BP scaffolds have excellent cellular biocompatibility.

#### 2.2.3 Polarization, Inflammatory Gene Expressions of RAW Cells on PLGA/BP Scaffolds

It is well known that the initial inflammatory phase is more important than the subsequent active bone repair phase and macrophages play a decisive role control the inflammatory response and maintaining bone homeostasis in bone regeneration.^[^ ^27^ ^]^ In fact, macrophages play a key role in controlling the immune response and tissue remodeling by inducing cytokines release and recruiting other immune cells to the implant site. Moreover, macrophages have strong plasticity, which can have a positive impact on the surrounding tissue microenvironment through the phenotypic switch, thereby promoting tissue regeneration.^[^ ^28^ ^]^

**Figure 3:**
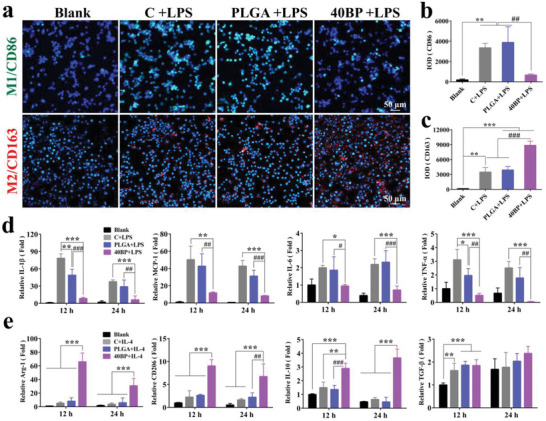
Macrophage polarization on PLAG/BP scaffolds. a) After RAW264.7 cells were seeded on the different scaffolds and stimulated with LPS for 24 h, the expression of M1 biomarker CD86 (green) and M2 biomarker CD163 (red) was detected by fluorescence staining. b) Quantification of the integrated optical fluorescence density (IOD) of CD86. c) Quantitative analysis of CD163. d) Inflammatory genes were detected in RAW264.7 cells with different treatments at 12 and 24 h by RT‐qPCR. e) M2 marker genes were detected in RAW264.7 cells with different treatments at 12 and 24 h by RT‐qPCR. ^*^ *p* < 0.05, ^**^ *p* < 0.01, and ^***^ *p* < 0.001 by one‐way ANOVA with Tukey's post hoc test, when PLGA and 40BP groups compared with the Control group; ^#^ *p* < 0.05, ^##^ *p* < 0.01, and ^###^ *p* < 0.001 by one‐way ANOVA with Tukey's post hoc test, when 40BP group compared with PLGA group. *n* = 3 for biological replicates.

First, the polarization of macrophages on different scaffolds was investigated in vitro. We qualitatively evaluated how different PLGA/BP scaffolds affected macrophage polarization in response to LPS stimulation using immunofluorescence staining and confocal microscopy. Typical surface markers of macrophages were differentially expressed in different genotypes, and CD86 was found to be strongly expressed in the proinflammatory macrophage stage (M1).^[^ ^29^ ^]^ The M2 phenotype, activated by the typical surface marker CD163, helps to downregulate inflammation and enhance tissue healing.^[^ ^30^ ^]^ RAW264.7 cells were cultured with 40BP scaffolds after 24 h, and the expression of CD86, a phenotypic marker identifying M1‐type macrophages, decreased significantly (*p* < 0.01) compared to other groups. On the other hand, the expression of CD163, a phenotypic marker identifying M2‐type macrophages, was significantly increased (*p* < 0.001, **Figure** **3**a–c) compared to other groups. Compared with the LPS‐stimulated control group, the fluorescence intensity of CD163 expression was similar in the PLGA group. This suggests that PLGA/BP scaffolds can promote the conversion of M1 to M2 type of macrophages. Then, to determine the concentration of BP that can suppress inflammation, we studied the effect of varying concentrations of BP on Raw264.7 cells (Figure S3, Supporting Information). In addition, anti‐inflammatory cytokines secretion was detected under the same culture conditions for 12 and 24 h to confirm the effect of the different materials on the cells. The inflammatory genes expressed in RAW264.7 cells by the RT‐PCR assay shown  that increased expression of TNF‐α, IL‐6, MCP‐1, and IL‐1β indicated LPS‐stimulated control group of inflammatory macrophages (M1‐like) activation (Figure 3d). In contrast, inflammatory gene expression was significantly downregulated in macrophages cultured with 40BP scaffolds (*p* < 0.05). TNF‐α and IL‐1β inhibited bone morphogenetic protein (BMP2) induced osteoblastic differentiation, and alkaline phosphatase activity,^[^ ^31^ ^]^ while IL‐6 also plays a key role in fracture healing during the early stages.^[^ ^32^ ^]^ We also investigated the effects of 40BP on the expression of M2 gene markers at different time points. As shown in Figure 3e, 40BP notably enhances the mRNA levels of M2 gene markers, including Arg‐1, CD206, IL‐10, and TGF‐β, at 12 and 24 h in IL‐4 treated RAW264.7 cells. These results suggest that PLGA/BP scaffolds can recruit macrophages and inhibit inflammation to stimulate the M2 polarization of macrophages and promote bone regeneration.

#### 2.2.4 Transcriptomic Analysis of the PLGA/BP Scaffolds for Osteogenic Differentiation

**Figure 4:**
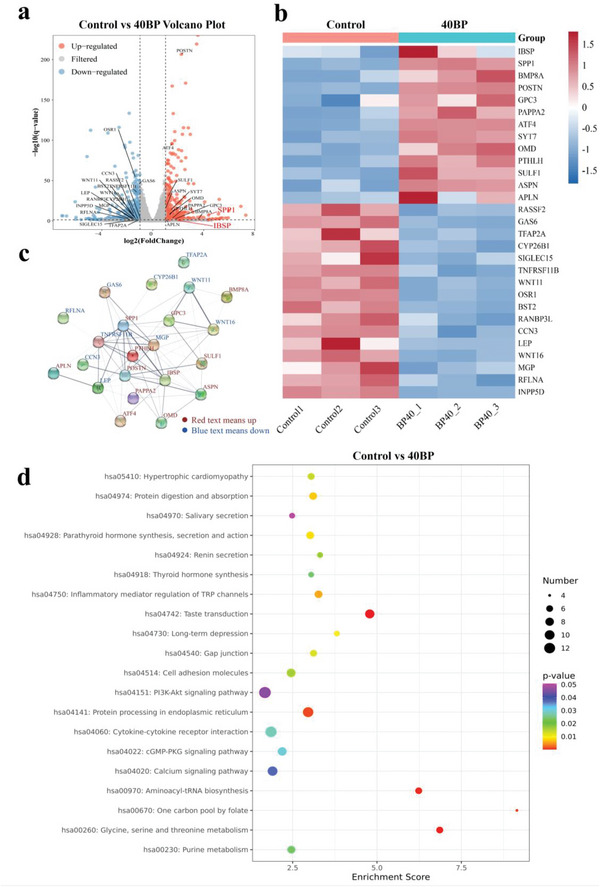
Transcriptomic analysis of osteogenic induction of PLGA/BP scaffolds. a) The volcano image of differential expression of osteogenic differentiation of hBMSC induced by osteogenic induction medium and 40BP scaffolds with leaching solution. Gray represents non‐significant gene differences, orange represents up‐regulated genes, and blue represents down‐regulated genes. b) The heat map of differential genes between the control and 40BP scaffold groups, red represents an up‐regulated gene, and blue represents the down‐regulated gene. c) The protein interaction network analysis of the differential genes based on the database STRING, red represents the upregulated gene, and blue represents the downregulated gene. d) The bubble map of KEGG enrichment analysis and the horizontal axis represents the enrichment score. *n* = 3 independent samples.

To further investigate the molecular mechanism of BP on osteogenic differentiation and mineralization, we performed transcriptomic analysis of hBMSC cultured on PLGA/BP scaffolds with degradation release leaching. A genome alignment of each sample was obtained by mapping clean reads to the reference genome, and the alignment rate for each sample ranged from 98.29% to 98.7%. This data was used to analyze gene expression levels in protein‐coding genes. By comparing the expression of protein‐coding genes among the samples, differential screening was performed, and there was a total of 6 differential groups. **Figure** **4**a is the volcano image of differential expression, in which gray are the genes with a nonsignificant difference, and orange and blue are the genes with a significant difference. Statistics of differentially expressed genes show 357 upregulated genes and 593 downregulated genes. Red represents an up‐regulated expression of protein‐coding genes and blue represents a down‐regulated expression of protein‐coding genes in the differential gene grouping cluster diagram (Figure 4b). Integrin‐binding sialoprotein (IBSP) was 3.9‐fold higher and osteopontin (secreted phosphoprotein 1, SPP1) was 20.8‐fold higher compared with the control group. Bone morphogenetic protein 8 (BMP8A) increased 11.8‐fold compared with the control group. Figure 4c is the diagram of protein interaction network analysis generated online from the STRING database. Specific gene heat maps and the corresponding protein‐protein interaction networks highlighted that the 40BP group could play a positive role in the recruitment of hBMSC by stimulating the expression and interaction with the osteogenic‐related proteins. The Kyoto encyclopedia of genes and genomes (KEGG) database was used for pathway analysis of DEGs (Figure 4d), and the enrichment score is represented on the horizontal axis. The element with a larger bubble contains more DEGs. The enrichment score of the PI3K‐AKT signaling pathway was the highest, suggesting that the 40BP group could accelerate osteogenesis through the PI3K‐AKT signaling pathway. In addition, the 40BP group significantly promoted cell adhesion molecules, protein processing in the endoplasmic reticulum, interaction between cytokine and cytokine receptors, calcium signaling pathway, cyclic guanosine monophosphate‐protein kinase G (cGMP‐PKG) signaling pathway, and regulation of inflammatory mediators of TRP channels. These results have identified which genes and signaling pathways BP promotes bone regeneration by activating or inhibiting.

In order to explore the molecular mechanism of BP in osteogenic differentiation and mineralization, we performed a transcriptomic analysis of hBMSC cultured on PLGA/BP scaffolds of degradation release leaching solution, as shown in Figure 4a,b. Multiple genes that are differentially highly expressed (e.g., IBSP, SPP1 BMP8A, etc.) all play an important regulatory role in osteogenic differentiation and were validated in vitro. Interestingly, during the transcriptome screen, we identified two bone matrix protein genes IBSP and SPP1, that were significantly upregulated by the 40BP group. It has been shown that IBSP and SPP1 are markedly upregulated in immature osteoblasts, and both were affected by Runt‐related transcription factor 2(Runx2).^[^ ^33^ ^]^ The results of this section (Figure 4c) suggest that BP is most likely to play an active role in bone matrix formation. Moreover, KEGG database analysis results (Figure 4d) show that cell adhesion molecules, protein processing in the endoplasmic reticulum, cGMP‐PKG signaling pathway, PI3K‐AKT signaling pathway, these signaling pathways related to tissue damage repair were significantly upregulated, and also PI3K‐AKT signaling pathway may contribute to maintaining the macrophage M2 polarization.^[^ ^34^ ^]^ In addition, the calcium signaling pathway is upregulated further proving that Ca^2+^ and phosphate work together to accelerate bone defect repair.

#### 2.2.5 Mechanism of Osteogenic Differentiation and Mineralization

**Figure 5:**
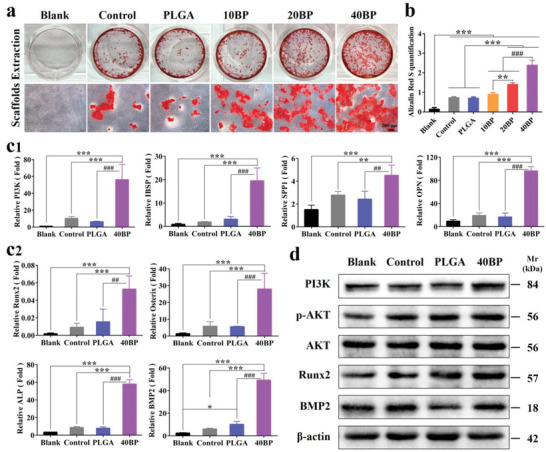
PLGA/BP scaffolds promote osteogenic differentiation and mineralization by activating PI3K‐AKT signaling pathway. hBMSC cells were cultured for 14 days with α‐MEM (blank group), osteogenic induction medium (control group), osteogenic induction medium with PLGA scaffold leaching solution (PLGA group), or different PLGA/BP scaffold leaching solutions (10BP, 20BP, and 40BP groups). a) Calcium deposition stained with Alizarin Red S after 14 days of cultivation. b) Quantitative analysis of alizarin red S staining. c) RT ‐qPCR analysis of transcriptome screening genes (**c1,** including PI3K, IBSP, SPP1, and OPN) and transcription factors related to osteogenic differentiation (**c2,** including Runx2, Osterix, ALP, and BMP2), genetic expression of hBMSC cells with different treatments for 14 days. d) Western blot assay of protein level of PI3K, p‐AKT, AKT, Runx2, and BMP2; β‐actin was used as a protein loading control. ^*^ *p* < 0.05, ^**^ *p* < 0.01, and ^***^ *p* < 0.01 by one‐way ANOVA with Tukey's post hoc test when PLGA and 40BP groups were compared with a control group; ^#^ *p* < 0.05, ^##^ *p* < 0.01, and ^###^ *p* < 0.001 by one‐way ANOVA with Tukey's post hoc test when 40BP group was compared with PLGA group. *n* = 3 for biological replicates.

The osteogenic differentiation levels of different PLGA/BP scaffold groups were assessed by semiquantitative analysis of calcium nodule staining. On day 14 of osteogenesis induction, it was observed that the hBMSC cells induced by the scaffold extract showed different degrees of deep red calcium accumulation in all experimental groups (**Figure** **5**a), especially the maximum calcium deposition density after the culture of 40BP extract (*p* < 0.001, Figure 5b), indicating that the 40BP scaffolds promote osteogenic differentiation more effectively. To further evaluate the expression of related genes during osteogenic differentiation, transcriptome‐related screening genes (PI3K, IBSP, SPP1, OPN, Figure 5c1) and osteogenesis‐related genes (Runx2, Osterix, ALP, BMP2, Figure 5c2) were detected and verified by RT‐PCR. PI3K is an intracellular phosphatidylinositol kinase that regulates cell proliferation, differentiation, migration, and glucose transport. Runx2, Osterix, and BMP2 were all activated via the PI3K‐AKT signaling pathway.^[^ ^35^ ^]^ Runx2 transcription factor is one of the key factors for osteoblast differentiation and bone formation. It mainly regulates the cell division cycle and interacts with other transcription factors during the differentiation process. During bone maturation, osteoblasts secrete proteins to form the extracellular matrix of bone, including alkaline phosphatase (ALP), BMP, and osteopontin (OPN). ALP allows calcium phosphate to accumulate in the extracellular matrix of bone, while bone morphogenetic protein and osteopontin affect systemic metabolism and bone mineralization. Moreover, the protein expression of PI3K and p‐AKT was up‐regulated in the 40BP group. For those functional proteins in osteogenesis, Runx2 expression of the 40BP group was also remarkably up‐regulated compared with Control and PLGA groups (Figure 5d). The result of transcriptomic analysis was consistent with osteogenic differentiation experimental results, suggesting that 40BP scaffolds could promote osteoblast protein secretion, osteogenic differentiation, and osteogenic behavior through PI3K‐AKT signaling pathway.

### 2.3 In Vivo Animal Studies

#### 2.3.1 In Vivo Assessments of the Immunomodulatory Properties of PLGA/BP Scaffolds

**Figure 6:**
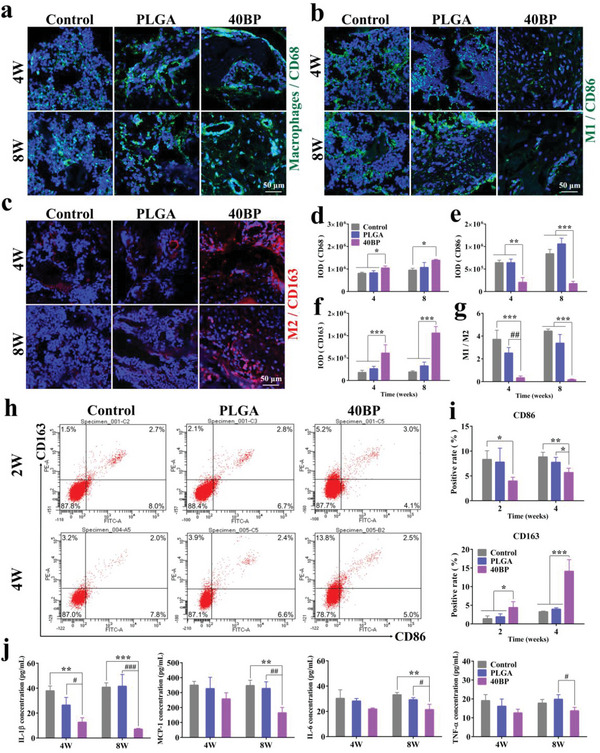
In vivo assessments of the immunomodulatory effect of PLGA/BP scaffolds in the distal femoral defect of the SAON rat. a) Immunofluorescence staining of CD68 for mononuclear macrophages in different groups. b) Immunofluorescence staining of CD86 for M1 macrophage phenotypes in different groups. c) Immunofluorescence staining of CD163 for M2 macrophage phenotypes in different groups. d–f) Quantitative analysis of the integrated optical density of CD68, CD86, and CD163 fluorescence in different groups at 4 and 8 weeks after surgery. g) The ratio of CD86/CD163 in the distal femoral defect area of the SAON rat 4 and 8 weeks after surgery. h) Measurement of the relative content of M1 and M2 subtypes of macrophages on PLGA and 40BP scaffolds implanted in rats for 2 and 4 weeks by flow cytometry. i) Quantification of the content of CD68‐ and CD163‐positive cells. j) The expression level of inflammatory factors in rat serum by ELISA. ^*^ *p* < 0.05, ^**^ *p* < 0.01, and ^***^ *p* < 0.001 by one‐way ANOVA with Tukey's post hoc test when PLGA and 40BP groups were compared with the control group; ^#^ *p* < 0.05, ^##^ *p* < 0.01, and ^###^ *p* < 0.001 by one‐way ANOVA with Tukey's post hoc test when 40BP group was compared with PLGA group. *n* = 3 for biological replicates.

Although our data show that BP induces M2 polarization of macrophages and inhibits inflammation to coordinate bone regeneration in vitro, this observation seems less convincing without in vivo animal studies to support it. Therefore, we further researched the immunomodulatory functions using the SAON rat model. We performed immunofluorescence staining of macrophages from the different experimental groups on rat femoral slices. **Figure** **6**a shows the expression of CD68 immunofluorescence in mononuclear macrophages of each group. The phenotype of M1 macrophages was identified with the CD86 antibody (green, Figure 6b), and the phenotype of M2 macrophages was identified with the CD163 antibody (red, Figure 6c). The statistical results showed that both PLGA and 40BP scaffolds could recruit M2 macrophage cells (Figure 6d). The expression level of CD86 immunofluorescence in the 40BP group was lower than the control and PLGA control groups significantly (*p* < 0.01, Figure 6e). The immunofluorescence of CD163 in the 40BP group was significantly higher than the blank and PLGA control groups (*p* < 0.001, Figure 6f). The immunofluorescence results further confirmed that PLGA/BP scaffolds could induce macrophage M1 (CD86) to M2 (CD163) polarization. The ratio of CD86/CD163 was significantly decreased in the 40BP group than in the blank and PLGA control groups (*p* < 0.01, Figure 6g). The results indicated that BP could regulate the osteoimmune environment around the implant site in rats. The 40BP group could effectively promote the phenotype of M2 macrophages and reduce the phenotype of M1 macrophages in vivo, which had a positive influence on the formation of new bone in the anti‐inflammatory local microenvironment.

At 2 and 4 weeks after surgery, flow cytometry (FCM) was used to detect CD86 and CD163 to measure the relative content of M1 and M2 cells with the defect areas in the control, PLGA, and 40BP groups. In Figure 6h, cells in the Q1 region were labeled with flow cytometry antibody CD163 and cells in the Q4 region were labeled with flow cytometry antibody CD86. Figure 6i is the result of data integration and analysis. The CD86 positive rate of the 40BP group was 3.9% (*p* < 0.05) and 2.8% (*p* < 0.01) lower than that in the control group, and 2.6% and 1.6% (*p* < 0.05) lower than that in PLGA group at 2 and 4 weeks, respectively. The CD163 positive rate in the 40BP group was 3.7% (*p* < 0.05) and 10.6% (*p* < 0.001) higher than that in the control group at 2 and 4 weeks, respectively, and 9.9% (*p* < 0.001) higher than that in PLGA group at 4 weeks. The immune‐related cytokines were examined in vivo by enzyme linked immunosorbent assay (ELISA) to further test the inflammatory cytokines secretion (Figure 6j). Compared to control and PLGA groups, TNF‐α, IL‐6, MCP‐1, and L‐1β were downregulated in the 40BP group, and this effect became more evident at 8 weeks than 4 weeks after the scaffold implantation. The ELISA test results of inflammatory factors from rat serum also demonstrate that PLGA/BP scaffolds inhibit pro‐inflammatory secretion in vivo. Both in vivo and in vitro results demonstrate that PLGA/BP scaffolds have modulated effect on the macrophages polarization and are able to inhibit the inflammatory response during the early stage of bone injury and create a favorable osteoimmune environment, facilitating the differentiation of bone marrow stem cells and subsequent bone regeneration.

Based on the fact that the effect of BP on macrophages in bone regeneration is unknown, it has been reported to suggest that BP may have an immune suppressive function.^[^ ^36^ ^]^ So, we focused on the effect of BP on macrophages in our study. In vitro, the immunofluorescence results in Figure 3a–c show the PLGA/BP scaffolds facilitate the macrophages M1(CD86) to M2(CD163) polarization, and inflammatory genes typically observed in M1 macrophages (e.g., TNF‐α, IL‐6, IL‐1β, and MCP‐1) were significantly downregulated (Figure 3d). TNF‐α and IL‐1β suppressed BMP‐2‐induced osteoblastic differentiation, and alkaline phosphatase activity,^[^ ^37^ ^]^ while IL‐6 is also most important in the early stages of fracture healing.^[^ ^32^ ^]^ These results suggest that PLGA/BP scaffolds can recruit macrophages and inhibit inflammation to stimulate macrophages M2 polarization and promote bone regeneration. Although our data demonstrated BP could induce macrophages M2 polarization and inhibits inflammation to coordinate bone regeneration in vitro, without in vivo animal studies to support this observation, it may seem less convincing. So, we used the SAON rat mode, an established nontraumatic osteonecrosis modle caused by high doses of steroids, which are induction composed of LPS and high doses of MPS.^[^ ^14^ ^]^ This animal model established by  injection of LPS resulted in an inducted inflammatory response, which could be used to detect the function of BP in inhibiting inflammation. We further explored the in vivo immunomodulatory effects. When PLGA and 40BP scaffolds are implanted after 2 weeks and 4 weeks in rat distal femur bone defect region, macrophages are recruited to the biomaterial surface and detected of M1 and M2 subtype by flow cytometry (Figure 3e). Macrophages switch M1 to M2 phenotype can be detected in the scaffolds after 2 weeks implantation. Furthermore, according to the immunofluorescence figures (Figure 6a–c), a higher proportion of M2 macrophages (CD163) and a lower ratio of M1 macrophages (CD86) were induced by PLGA/BP scaffolds, indicating that the elevated M2/M1 ratio in bone defect region. The ELISA test results from in rat serum of inflammatory factors further prove PLGA/BP scaffolds inhibit pro‐inflammatory secretion in vivo (Figure 6j). The results in vitro and in vivo reveal that the PLGA/BP scaffolds can modulate macrophage M1 switch to M2 polarization and inhibit the early‐stage inflammatory response in bone injury region, and generate a favorable osteoimmune environment, thereby promoting the BMSCs differentiation and subsequent bone regeneration.

#### 2.3.2 Analysis of New Bone Formation by Micro‐CT

**Figure 7:**
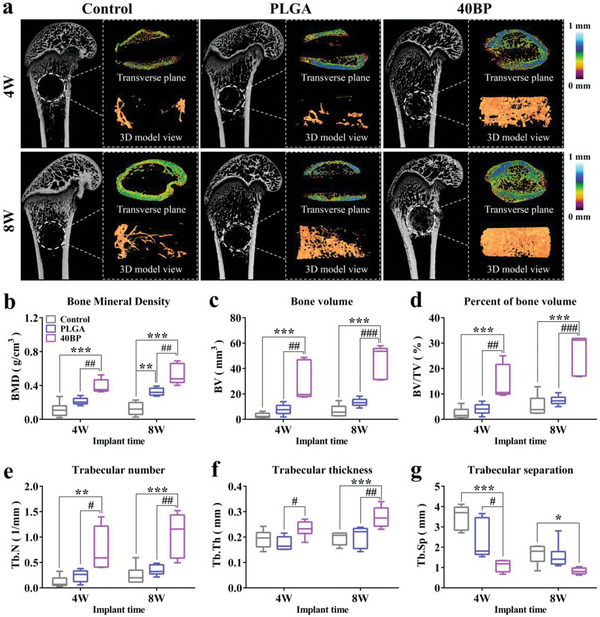
In vivo analysis of the new bone formation of PLGA/BP scaffolds in the distal femoral defect of the SAON rat using micro‐CT. a) Micro‐CT 3D reconstruction images of the representative region of interest (ROI) in the area of the bone defect of the distal femur in different groups 4 and 8 weeks after surgery. The distribution of trabecular thickness was shown in the transverse plane. b–g) Quantitative analysis results of micro‐CT of new trabecular bone in the bone defect area 4 and 8 weeks after surgery: b) BMD; c) BV; d) BV/TV; e) Tb.N; f) Tb.Th; g) Tb.Sp. ^*^ *p* < 0.05, ^**^ *p* < 0.01, and ^***^ *p* < 0.001 by one‐way ANOVA with Tukey's post hoc test when PLGA and 40BP groups were compared with the control group; ^#^ *p* < 0.05, ^##^ *p* < 0.01, and ^###^ *p* < 0.001 by one‐way ANOVA with Tukey's post hoc test when 40BP group was compared with PLGA group. *n* = 6 for biological replicates.

The osteogenesis effect of the 40BP scaffolds was evaluated by implanting the scaffold into the distal femoraldefects in the SAON rat models. At 4 and 8 weeks after surgery, the bone defect site was scanned by micro‐CT and reconstructed for analyzation (**Figure** **7**a). The scaffolds in each group did not loosen or fall off, and new bone tissue began to grow into the defect cavity at 4 weeks after surgery. The 2D image on the transverse plane showed bone structure and distribution of trabecular thickness. The thickness of bone trabeculae newly generated in the defect area in the 40BP group was increased than the control group and PLGA group, and the bone thickness in the cortical bone area in the 40BP group was thicker than the control group. The 3D reconstruction image of the new bone also suggested that the repair effect of 40BP scaffolds was more impressive than that in the control and PLGA groups (coronal plane). At 8 weeks after surgery, the internal and external new bone of the surgical cavity in the empty control group and PLGA group gradually increased, while, unlike the 40BP group, it was still not fully restored. The quantitative analysis of bone growth obtained from the defect ROI of the surgical cavity in the experimental groups is shown in Figure 7b–g, including bone mineral density (BMD), bone volume (BV), percentage of bone volume (BV/TV), and trabecular analysis of bone. The BMD, BV, and BV/TV were significantly higher in the 40BP group than the control (*p* < 0.001) group and PLGA group (*p* < 0.01) at both 4 and 8 weeks after surgery. Also, the quantitative analysis of bone trabecular in the 40BP group exhibited a significant improvement. At 4 weeks after surgery, the trabeclar number (Tb.N) was significantly higher in the 40BP group than in the control (*p* < 0.01) and PLGA group (*p* < 0.05). Also, the trabeclar thickness (Tb.Th) was a significant increase compared to the PLGA group (*p* < 0.05). For the trabeclar separation (Tb.Sp), its decline in the 40BP group suggested a porosity increase in new bone, which further indicated the repairment of trabecular bone. A similar trend was observed at 8 weeks after surgery, with higher significance in the Tb.N and Tb.Th of the 40BP group when compared to the control (*p* < 0.001) and PLGA group (*p* < 0.01). With tissue restoration, the Tb.sp value tends to be the same, but there is still a significant difference between 40BP and the control (*p* < 0.05). Above all, the BP scaffolds exhibit excellent osteogenesis and bone integration ability, proving that the osteoimmune microenvironment promotes osteogenesis and bone regeneration process.

To further illustrate the effect of osteoimmune microenvironment induced by PLGA/BP scaffolds on osteogenesis, the osteogenic differentiation levels of PLGA/BP scaffolds with different concentrations were evaluated by calcium nodular staining (Figure 5a,b). The results of staining showed that BP had a good gradient effect on osteogenic differentiation, and 40BP showed stronger ability to promote osteogenic differentiation. Also, Runx2, Osteocalcin (OCN), ALP, BMP2, etc., osteogenic differentiation‐related transcription factors were detected by Q‐PCR and verified upregulated significantly. In vivo, SAON rat distal femur bone defect model was used to further assess the effects in bone regeneration, and 3D reconstruction by micro‐CT scans of bone defect analysis as shown in Figure 7a. The bone defect was well repaired at 4 weeks and almost completely repaired at 8 weeks after implanted. Furthermore, the PLGA/BP group showed improved BMD, BV/TV, and Tb.Th and Tb.N of new bone tissue (Figure 7b–g). PLGA/BP scaffolds exhibit excellent osteogenesis and bone regeneration and effectively prove that osteoimmune microenvironment promotes osteogenesis and bone regeneration process.

#### 2.3.3 Histological Analysis

**Figure 8:**
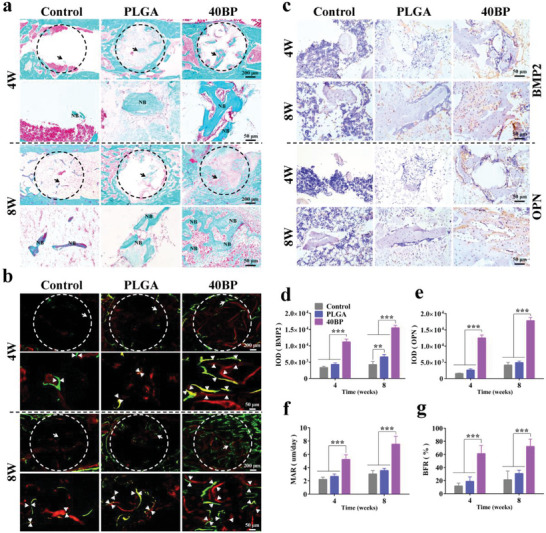
Histological analysis of new bone formation in the area of the distal femoral defect of the SAON rat at 4 and 8 weeks after surgery. a) Goldner's trichrome staining of the bone defect area of the different groups. The black circle indicates the bone defect tunnel at 40× magnification; the site of new bone formation indicated by the black arrow is shown below at 200× magnification. b) Sequential fluorescence micrographs of the undecalcified sections at 4 and 8 weeks. Green and red fluorescence is from calcein‐AM and xylenol orange excited at 480 and 560 nm, respectively. White double arrows indicate the distances between the new bone tissue. c) Immunohistochemical staining of BMP2 and OPN in the control, PLGA, and 40BP groups to evaluate bone regeneration in the defect area. d,e) Quantitative analysis of BMP2 and OPN based on the immunohistochemical images. f,g) Quantitative analysis of bone regeneration based on sequential fluorescence images. ^*^ *p* < 0.05 and ^**^ *p* < 0.01 by one‐way ANOVA with Tukey's post hoc test. *n* = 3 for biological replicates.

To evaluate the growth of new bone tissue and the integration effect of the bone‐implant interface, Goldner trichrome staining (**Figure** **8**a) and H&E staining (Figure S4b, Supporting Information) were performed in the control, PLGA, and 40BP groups. After 4 weeks of LPS hormone‐induced osteonecrosis, we extracted mesenchymal stem cells from the femoral bone marrow lumen of the model (rBMSCs) and found that most of them differentiated toward adipocytes (Figure S4a, Supporting Information), representing the successful establishment of the SAON rat model. In the control group, the bone defect areas were mainly filled with fibrous tissue and showed little new bone formation at 4 and 8 weeks after surgery. In the PLGA group, only a few signs of bone‐like tissue in the bone defect area. However, in the 40BP group, numerous mineralized bone tissue (green) was detected by staining. In order to further analyze bone regeneration, proteins associated with osteogenesis (BMP2, OPN, and OCN, Figure 8c; Figure S4c, Supporting Information) were detected by immunohistochemical staining. BMP2 is a transcription factor that plays important roles in bone and cartilage development, and OPN could promote aggregation of hydroxyapatite (HA) to form a crystal core. At 4 and 8 weeks after the scaffold implantation, the expression of the 40BP protein associated with osteogenesis was increased remarkably than in the control and PLGA groups (Figure 8d,e), indicating the excellent bone regeneration capacity of the 40BP scaffolds. In order to investigate the effect of new bone formation and remodeling, mineralized bone tissue was labeled with sequential fluorescent labeling at different time points. Using the fluorescence images (Figure 8b), we performed quantitative analysis and found that the mineral deposition rate (MAR, *p* < 0.001, Figure 8f) and bone formation rate (BFR, *p* < 0.001, Figure 8g) significantly increased in the 40BP group than in the control and PLGA groups at 4 and 8 weeks after surgery, demonstrating that bone tissue formation and repair were accelerated after implantation of 40BP scaffolds. These histological data demonstrate that 40BP scaffolds could significantly improve the formation of new bone and achieve an effective treatment effect on bone defects.

## 3 Conclusion

3D printing of porous PLGA/BP scaffolds was used to investigate the osteoimmunomodulatory effects of BP on the bone tissue microenvironment and even subsequent bone regeneration. The porous PLGA/BP scaffolds can remarkably suppress the inflammatory cytokines and promote macrophage polarization to the M2 type. In vivo, this pro‐osteogenic microenvironment promotes the proliferation and differentiation of hBMSC by upregulating IBSP, SPP1, ALP, Osterix, OPN, R2, and BMP2, which further accelerates bone regeneration. Moreover, PLGA/BP scaffolds have excellent osteogenesis, biocompatibility properties, and mechanical properties, and could effectively promote bone regeneration through the PI3K‐AKT signaling pathway. Therefore, PLGA/BP scaffolds are an excellent bone substitute that can be used in the treatment of bone defects with immunomodulatory in situ bone and osteogenesis.

## 4 Experimental Section

### Materials and Scaffolds Fabrication

The BP nanosheet dispersion was purchased from Shenzhen Zhongke Mo Phosphorus Technology Co, Ltd (Guangdong, China) and stored in N‐methyl‐2‐pyrrolidone (NMP) (99.5%, anhydrous). PLGA (medical grade, lactic acid to glycolic acid ratio 75:25, molecular weight 300 kD) was obtained from the Shangdong Institute of Medical Instruments (Jinan, China). 1,4‐Dioxane was purchased from Shanghai LingFeng Chemical Reagent Co, Ltd (Shanghai, China). Mimics 20.0 software was used to design the unit structure of the carrier, and the Standard Mosaic Language (STL) format was adopted. The designed unit structure was then entered into the STL data to create a CAD model of the cylindrical beam with a 15 mm diameter and 18 mm height.^[^ ^38^ ^]^ Through micro‐CT software to revise the 3D printing pore geometry. Before molding, the concentration of dispersion of BP nanosheets was measured using a UV spectrophotometer (UV‐3600, Sinotech Import & Export Shenzhen Co., Ltd., Guangzhou, China). The dispersion was centrifuged for 10 min at 7000 rpm, and the BPQDs supernatant was carefully decanted and then resuspended in 1,4‐dioxane. Various amounts of BP nanosheets were added to a 1,4‐dioxane solution containing 15 wt.% PLGA. Using −30 °C low‐temperature 3D printing technology (CLRF‐2000‐II, Tsinghua University, Beijing, China), the polymer scaffolds were prepared with different contents of BP nanosheets of 0 wt.% (PLGA), 0.01 wt.% (10BP), 0.02 wt.% (20BP), and 0.04 wt.% (40BP). After preparation, the scaffolds were freeze‐dried in a freeze‐drying unit (Alpha 2–4 LDplus, Marin Christ, Germany) for 48 h, then dried at 37 °C for 7 days in a vacuum oven to completely remove the solvent 1,4‐dioxane.^[^ ^39^ ^]^ The surface morphology, pore size, and micropore morphology of the four groups of scaffolds were examined by scanning electron microscope (SEM, Zeiss Supra55, Carl Zeiss, Germany). Meanwhile, energy‐dispersive X‐ray spectroscopy (EDS, X‐Max 20, Oxford, UK) was used to analyze the distribution of elements and the purity of the components of the 3D‐printed scaffolds. For the BP nanosheets alone, after dispersing them on an ultrathin carbon film, high‐resolution transmission electron microscopy (TEM, FEI Tecnai G2 F20 S‐Twin 200 kV, USA) was performed to reveal the structural morphology and lattice composition of the crystal. After the scaffolds were crushed into powder at low temperature using a multi‐sample tissue crusher ( JingXin, Tissuelyser‐24, Shanghai, China), Raman spectroscopy (Raman, Thermo Fisher, DXR2xi, USA), X‐ray diffraction (XRD, Bruker, D8 advance, Germany), and Fourier transform infrared spectroscopy (FT‐IR, Thermo Fisher, Nicolet IS5,USA) were performed to analyze the structure of the BP after 3D printing. The elemental composition and valence state of BP scaffolds were analyzed by X‐ray photoelectron spectroscopy (XPS, Thermo Scientific, ESCALAB XI, USA).

### Scaffolds Degradation

The standard for degradation of the scaffolds was referred to ISO 10993‐13:2010. Scaffolds were immersed in PBS buffer solution (pH 7.4) at 37 °C at a volume ratio of 0.1 g mL^−1^, and the supernatant was collected after 0, 7, 14, and 28 days. After acidolysis with nitric acid, ICP‐OES (700 Series, Agilent Technologies, USA) was used to determine the release concentration of P in the immersion solution at the respective time points. The weight loss percentage of the scaffold for different degradation times (0–28 days) was determined by the equation: Weight loss (%) = (*W*_0_‐*W*r)/*W*_0_×100 (*W*_0_: original weight, *W*r: remaining dried weight). The mechanical properties of the Scaffolds were tested and measured using a static and dynamic materials testing machine (Instron‐E3000, Norwood, MA, USA). The testing speed was set at 1 mm min^−1^, and four specimens were tested in each group to obtain the force‐displacement curve. The force‐displacement curve was used to calculate the compressive strength and compressive modulus of the beam according to the method of ISO 844:2004 standard.

### Biocompatibility Testing of BP Scaffolds

Human bone marrow mesenchymal stem cells (hBMSC, ATCC PCS‐500‐012) were seeded onto the scaffold surface, and cell proliferation was detected after 1, 3, and 7 days, respectively, using CCK‐8 reagent. The relative proliferation rate (RGR) of the cells was calculated based on the measurement results of the microplate meter. After 7 days of culture on the scaffolds, cells were fixed with 4% paraformaldehyde for 30 min, followed by 0.1% Triton 100 (Amresco, 0694) treatment for 5 min. Then, an appropriate amount of rhodamine‐labeled gholiptide (Abcam, ab176756) and DAPI (Beyotime Biotechnology, C1005) was co‐incubated with the cells on the scaffold surface for 30 min. The morphology and number of nuclei (blue, 405 nm) and actin (red, 555 nm) were observed using LSCM (ZEISS LSM900, Germany). For the cytotoxicity of the scaffold extracts, the experiment was performed according to GB/T16886.5‐2017.

### Cell Polarization and Gene Expression on BP Scaffolds

An immunofluorescence assay monitored the expression of CD163 and CD86 on RAW264.7 to assess macrophage polarization in vitro. The prepared groups of material samples were first sterilized and placed in a 12‐well plate. Each well was inoculated with 40 × 10^4^ RAW264.7 cells and incubated overnight, and culture was continued with medium containing 100 ng mL^−1^ lipopolysaccharide (LPS, Sigma–Aldrich, USA) for 12 h. CD163 (Biorbyt, ORB544646) and CD86 (Biorbyt, Orb500820‐cf594) were used to evaluate the polarization of RAW264.7 cells to the samples by immunofluorescence assay. The total RNA of the cells was collected after 24 h of growth. The mRNA was extracted following the RNA assay kit instructions (Axygen, AP‐MN‐MS‐11 RNA‐250), and cDNA was synthesized from the total mRNA.^[^ ^40^ ^]^ Target genes were amplified with SYBR Premix Ex Taq II (Takara, RR820B), cDNA template, and specific primers (Table S1, Supporting Information) using a Light Cycler 96 instrument (Roche, USA). All primers were designed and synthesized by Shanghai Sangon Bioengineering Technology Service Co. LTD.

### Biomineralization Experiment at BP Scaffolds

After a 3‐day seeding of 5 × 10^4^ hBMSC cells, the cells were treated with α‐MEM complete medium containing 10 mmol L^−1^ sodium‐β‐glycerol phosphate, 50 µg mL^−1^ vitamin C, and 10 nmol L^−1^ dexamethasone to induce osteogenesis. The hBMSC cells without induced differentiation were set as a blank control group. hBMSC that was induced but not administered was set as the control group, and extracts of various scaffolds were added to the other experimental groups. After 14 days of microscopic observation, a large number of calcium nodules was formed. The nodules were fixed for 30 min at room temperature by adding 10% neutral formalin solution to each well and stained for 30 min with 1% alizarin red (Sigma–Aldrich, A5533). After rinsing with ultrapure water, the solution was photographed and viewed under a microscope. The solution was dissolved with 10% hexapylpyridine (Sigma–Aldrich, 17 692 378) at room temperature and protected from light for 30 min. For semiquantitative analysis, the absorbance value (OD) was measured at 562 nm. At the time of osteogenic culture induction for 7 days, RNA was extracted with a kit (Axygen, AP‐MN‐MS‐RNA‐250) and then transcribed into cDNA using the instructions of the total RNA Miniprep Kit (Takara, RR036A). A 1 µL cDNA template for each sample was amplified in a 10 µL reaction system of using the real‐time PCR primers listed in Table S1 (Supporting Information). The osteogenesis protein was extracted from hBMSC by radio‐immunoprecipitation assay (RIPA) buffer for 7 days. Equal amounts of protein were loaded in appropriate concentrations of sodium dodecyl sulfate (SDS)–polyacrylamide gel electrophoresis and transferred into a polyvinylidene fluoride (PVDF) membrane. The detailed protocols of western blotting followed the previous study.^[^ ^38^ ^]^ The antibodies were obtained from Abcam, including Anti‐PI 3 Kinase p85 alpha (ab182651), anti‐protein kinase B (AKT, ab32505), anti‐p‐AKT (S473) (ab66138), Runx2 (ab76956), BMP2 (ab6285), β‐actin (ab8226), goat anti‐mouse IgG H&L (HRP)(ab6789), and goat anti‐rabbit IgG H&L (HRP)(ab6721).

### Transcriptome Sequencing and Differential Gene Expression Analysis

hBMSC (12 × 10^6^ cells mL^−1^) was cultured in 6‐well plates for 3 days. Then, the medium was changed with α‐MEM complete medium containing 10 mmol L^−1^ β‐glycerophosphate disodium salt (Sigma–Aldrich, G9422), 10 nmol L^−1^ dexamethasone (Sigma–Aldrich, D4902), 50 µg mL^−1^ ascorbic acid (Sigma–Aldrich, A4544), and leaching solution of the scaffolds. After 7 days of differentiation, cells were lysed with Trizol reagent (Life, 15 596 026), and cell lysates were stored at 80 °C before sequencing. Cufflinks were used to calculate the FPKM for each gene, and HTSeqcount was used to determine the number of reads for each gene. The R package DESeq (2012) was used for differential expression analysis. The threshold for significant differential expression was set at foldchange>2 (*p* < 0.05). Gene expression patterns were determined using hierarchical cluster analysis of differentially expressed genes (DEGs). The hypergeometric distribution was used to analyze Gene Ontology enrichment and KEGG pathway enrichment of DEGs. Utilizing Cuffcompare software, new transcripts were identified by comparing the reference genome and known annotated genes following the readout by String Tie. OE Biotech Co, Ltd (Shanghai, China) performed the transcriptome sequencing and analysis.

### SAON Rat Model and Scaffolds Implantation

Six weeks old male Sprague‐Dawley rats were obtained from Guangdong Medical Laboratory Animal Center (Guangzhou, China) and kept in a pathogen‐free facility at the Shenzhen Institute of Advanced Technology (Shenzhen, China). The animal experiment protocol was approved by the Research Ethics Committee of Shenzhen Institute of Advanced Technology (SIAT‐IACUC‐190312‐YGS‐LYX‐A0716). Briefly, an intraperitoneal injection of 10 mg kg^−1^ LPS was performed twice with an interval of 24 h. After 1 day, they received three continuous intramuscular injections of MPS (Pfizer, USA) at 24 h intervals. After 2 weeks of SAON induction, rats were anesthetized with isoflurane gas. The skin was incised bilaterally, and muscles were bluntly dissected to expose the distal femoral condyles, into which an electric drill was used to drill a tunnel with a diameter of 2.8 mm and a depth of 8 mm. The appropriate scaffolds were implanted in the tunnel. Three groups of subjects were randomly selected: Control, PLGA, and 40BP (*n* = 6 for each time point). After surgery, the experimental rats received intraperitoneal penicillin injection (100 000 UI penicillin solution/kg body weight) to prevent infections. The experimental rats were anesthetized with isoflurane gas 4 and 8 weeks after surgery. The femoral condyle was isolated for micro‐CT, histological analysis, and macrophage polarization assay.

### Cytokines Secretion and Macrophage Polarization in the SAON Rat Model

To evaluate the function of the implanted scaffolds in macrophage polarization in the different groups, immunofluorescence staining was performed. After 4 and 8 weeks, femoral specimens with bone defects were collected and decalcified with saturated EDTA solution, dehydrated with a graded ethanol solution, and embedded in paraffin wax. The 5 µm thickness of each section was used in this experiment. For immunofluorescence, CD68 (Abcam, ab125212), a cytoplasmic glycoprotein, is the most reliable marker for macrophages. To identify the M1/M2 subtype, the antibodies CD86 (Biolegend, 200 305) and CD163 (Biorbyt, orb544646) were chosen. After the paraffin sections were hydrated with an ethanol‐water solution, they were incubated with diluted CD68 (1:500), CD86 (1:200), and CD163 (1:200) antibody solutions overnight at 4 °C. After another wash step with the wash buffer, the corresponding secondary antibody was incubated with the paraffin sections for 1 h at room temperature. DAPI (Beyotime Biotechnology, C1005) was stained with the paraffin sections for 10 min. Sections were then observed with LSCM (ZEISS, LSM900) and the intensity of fluorescence was quantified with the software Image Pro Plus 6.0. Peripheral blood from mice in each group was collected at 4 and 8 weeks. Serum was carefully collected for analysis of anti‐inflammatory factors using the ELISA kit IL‐1β (Dakewe, 1 310 122), the MCP‐1 kit (Biosharp, BSER‐034), the IL‐6 kit (Dakewe, 1 310 602), and the TNF‐α kit (Dakewe, 1 317 202) according to the protocol. Optical density was measured using a microplate reader (Thermo Fisher Scientific, USA), and released concentrations were calculated using standard curves. All samples were analyzed in triplicate.

### Micro‐CT Analysis

After implantation for 4 and 8 weeks, SAON rats were killed under isoflurane general anesthesia. The femur samples were collected and scanned with high‐resolution micro‐computed tomography (Micro‐CT, Bruker, SkyScan 1176, Germany). Micro‐CT was performed to evaluate the bone parameters of the bone defect area at a voltage of 60 kV, a current of 417 µA, and a resolution of 18 µm with two average images and an angular step of 0.5°. Tomographic reconstruction data were then acquired using NRecon software, for which the parameters smoothing = 2, ring artifact correction = 5, and beam hardening correction = 40% were set. On this basis, a region of interest (ROI) was selected to analyze new bone formation in the area of the 2.8 mm diameter bone defect. Parameters reflecting bone quantity and quality were analyzed with CT‐Analyzer software. The test included BMD, BV, BV/TV, Tb.N), Tb.Th, and Tb.Sp. The 3D reconstructed micro‐CT images and the 3D model of the new bone tissue in the defect area were created using CTvol software.

### Histological Analysis

Femur specimens were decalcified with 10% EDTA (Boster Biological Technology Co. Ltd, AR1071) for 8 weeks at 37 °C, then dehydrated with a graded ethanol solution and embedded in kerosene wax. The 5 µm thickness of each section was used in this experiment. After rehydrating the sections with a series of 100%, 90%, 80%, and 70% ethanol solutions and a water solution, two additional experiments were performed: Goldner's trichrome staining and immunohistochemical staining. Goldner's trichrome staining (Sigma–Aldrich, HT10316) was performed according to the instructions of the kit. Sections were stained with hematoxylin (Sigma–Aldrich, 0 3971) for 20 min and then rinsed with 90% and 100% ethanol. Sections were immersed in xylene and sealed with resin. For immunohistochemical staining, BMP2 (Abcam, ab6285), OPN (Abcam, ab63856), and OCN (Santa Cruz Biotechnology, sc‐365797 HRP) were selected to evaluate bone formation. After the antigen in the sections was removed with citrate buffer (Beyotime Biotechnology, P0088) for 30 min at 95 °C, then incubated for 10 min with 3% hydrogen peroxide and blocked for 1 h with 5% goat serum at room temperature. After rinsing with water, the diluted BMP2 (1:1000), OPN (1:200), or OCN (1:50) antibody solutions were co‐incubated with the sections overnight at 4 °C. Following this, the secondary antibodies were incubated at room temperature for 1 h with the slides the following day. Then, the DAB solution (Abcam, ab64238) was dropped onto the sections to visualize the color of the antibody staining, and hematoxylin was used to visualize the nuclei. After dehydration of the tissue sections by alcohol solutions, the slides were mounted with resin. Finally, all mounted slides were viewed with a light microscope (Leica, DMi8, Germany), and fluorescence intensity was analyzed with Image Pro Plus 6.0 software. Mineralized bone tissue and new bone formation rate were detected by sequential fluorescent labeling. Calcein‐AM (10 mg kg^−1^, Sigma–Aldrich, 17 783) was injected subcutaneously twice into the different scaffold‐implanted rat groups 13 days before killing, whereas Xylenol Orange (90 mg kg^−1^, Sigma–Aldrich, 52 097) was injected subcutaneously 10 days apart. After killing the rats by isoflurane anesthesia, thigh samples were collected and fixed with 10% formalin. They were then dehydrated with 70%, 80%, 95%, and 100% ethanol, treated transparently with acetone for 1 day, and infiltrated with methyl methacrylate (MMA, Aladdin, M109623) for 3 days. During these processes, specimens were aspirated twice daily. Finally, the bone tissue was embedded with MMA in the absence of oxygen. After cutting, trimming, polishing, and photographing these embedded hard tissues, the green fluorescence of calcein was observed at 480 nm and the red fluorescence of xylenolorange was observed at an excitation wavelength of 560 nm. The white double arrow indicates the distance between new bone formation. Mineral deposition rate (MAR, µm per day) and bone formation rate (BFR, %) were calculated by double fluorescence staining.

### Statistical Analysis

GraphPad Prism 7 software (La Jolla, CA, USA) was used for statistical analysis. Quantitative data were presented as mean ± standard deviation. The statistical significance of pairwise comparisons with different groups was tested using the one‐way method ANOVA. Statistical significance was defined as *p* < 0.05.

## Conflict of Interest

The authors declare no conflict of interest.

## Author Contributions

J.L. conceived the project, executed all experiments, and wrote the manuscript. Z.Y.Y. designed the experiments and wrote the manuscript. W.Z. provided data interpretation and manuscript revisions. B.L. performed in vivo experiments and tissue analysis. K.M.C., X.F.D., and C.R.L. assisted with animal studies and experimental measurements. B.T. assisted with data analysis and interpretation. L.L. provided research guidance. L.Q. and X.F.Y. provided research guidance and revisions. Y.X.L. supervised the project and manuscript revisions. All authors approved the final version of the paper for submission. J.L. and Z.Y. contributed equally to this work.

## Supporting information

Supporting Information

## Data Availability

The data that support the findings of this study are available from the corresponding author upon reasonable request.
