# Supplementary material for: Regulation of Osteoimmune Microenvironment and Osteogenesis by 3D‐Printed PLAG/black Phosphorus Scaffolds for Bone Regeneration
Source: Adv Sci (Weinh). 2023 Aug 24;10(28):2302539. doi: 10.1002/advs.202302539 (PMC10558667; doi:10.1002/advs.202302539)
Supplement: Supplementary file 1 — Supporting Information [file ADVS-10-2302539-s001.pdf]

ADVANCED SCIENCE
Open Access

Supporting Information

for *Adv. Sci.*, DOI 10.1002/advs.202302539

Regulation of Osteoimmune Microenvironment and Osteogenesis by 3D-Printed PLAG/black Phosphorus Scaffolds for Bone Regeneration

*Jing Long, Zhenyu Yao, Wei Zhang, Ben Liu, Kaiming Chen, Long Li, Bin Teng, Xiang-Fu Du, Cairong Li, Xue-Feng Yu, Ling Qin and Yuxiao Lai**

Supporting Information

Regulation of osteoimmune microenvironment and osteogenesis by 3D-printed PLAG/black phosphorus scaffolds for bone regeneration

Jing Long[1, #], Zhenyu Yao[1, #], Wei Zhang[1], Ben Liu[1], Kaiming Chen[1], Long Li[1], Bin Teng[3], Xiang−Fu Du[1], Cairong Li[1], Xue-Feng Yu[4], Ling Qin[1,5,6], Yuxiao Lai[1,2,6,7, *]

Supplementary data

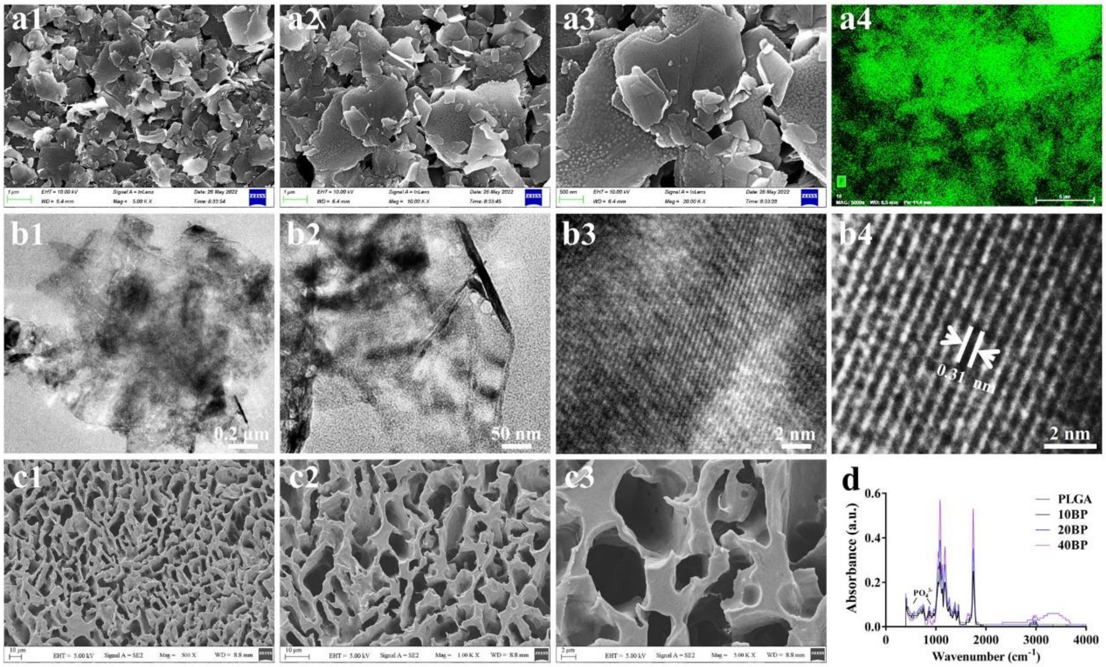

Supplementary Fig. 1. structural characterization of BP nanosheets and PLGA/BP scaffold. a1-a3. Morphological observation of BP nanosheets in different magnifications of SEM. **a4.** SEM-EDS for the phosphorus element of BP nanosheets. **b1-b4.** Observation of D-spacing of BP nanosheets at high resolution TEM. **c1-c3.** Observation of the morphology of PLGA/BP scaffolds by SEM at different magnifications, c1. 500×; c2.1000×; and c3. 3000×. **d.** The infrared absorption spectrum of PLGA/BP scaffolds. n=3 independent samples, * $p<0.05$, ** $p<0.01$, *** $p<0.001$ by one-way ANOVA with Tukey's post hoc test. n=3 independent samples.

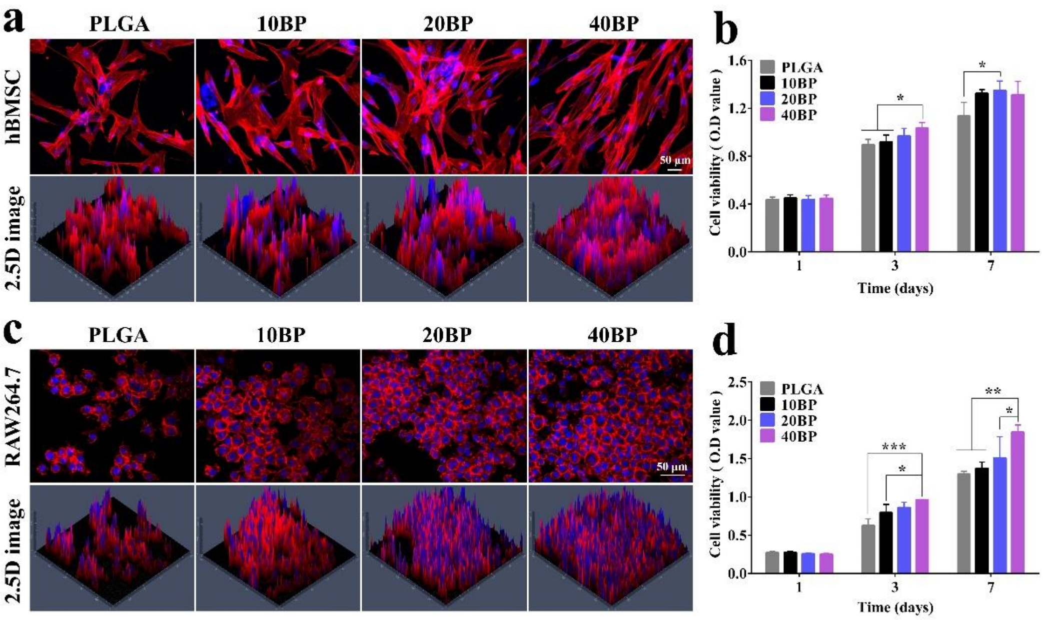

Supplementary Fig. 2. viability of hBMSC cells in the leaching solution of scaffolds of different BP concentrations. a. CLSM images of hBMSC cells stained with phalloidin (red) and DAPI (blue) after being cultured in the leaching solution of the scaffolds for 3 days. The 2.5D images show the distribution of fluorescence intensity. **b.** Relative cell viability of hBMSC cells cultured in the leaching solution of different scaffolds for 1, 3 and 7 days. **c.** CLSM images of RAW264.7 cells stained with phalloidin (red) and DAPI (blue) after being cultured in the leaching solution of the scaffolds for 3 days. **d.** Relative cell viability of RAW264.7 cells cultured in the leaching solution of different scaffolds for 1, 3 and 7 days. n=3 independent samples, * $p<0.05$, ** $p<0.01$, *** $p<0.001$ by one-way ANOVA with Tukey's post hoc test.

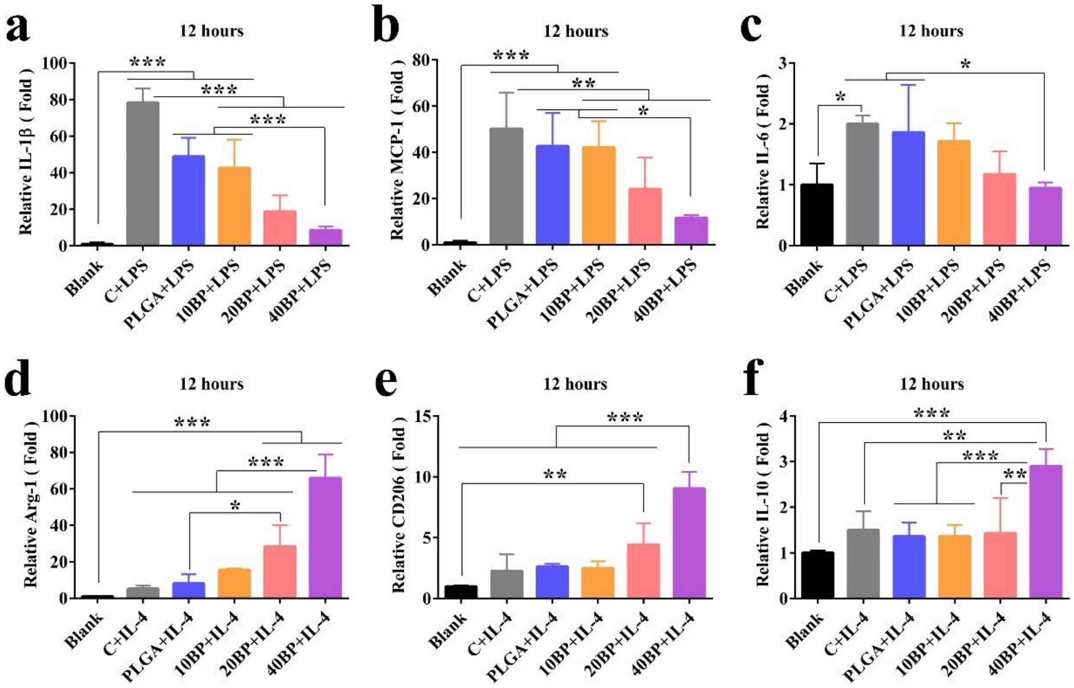

Supplementary Fig. 3. BP induce Macrophage polarization on Raw264.7 cells. After RAW264.7 cells were seeded on the different scaffolds and stimulated with LPS/IL-4 for 12 hours, the expression of M1 biomarker IL-6, MCP-1, IL-1β and M2 biomarker Arg-1, CD206 and IL-10 was detected by RT-qPCR with different concentrations of BPs. * $p<0.05$, ** $p<0.01$, *** $p<0.001$ by one-way ANOVA with Tukey's post hoc test. n=3 for biological replicates.

Isolation of rat BMSCs.

Rat BMSCs were isolated from the thighs of 2-week-old Sprague-Dawley rats (n=2, provided by Guangdong Medical Laboratory Animal Center) according to previous literature. Dulbecco's modified Eagle's medium (DMEM) supplemented with 10% fetal bovine serum (FBS) (Gibco, USA) containing bone marrow cells was cultured at 37°C and 5%CO_2 in a cell culture incubator. The medium was replaced for the first time after 24-48 hours and renewed every 3 days thereafter. Passage 3-5 (P3-P5) cells were used for subsequent experiments.

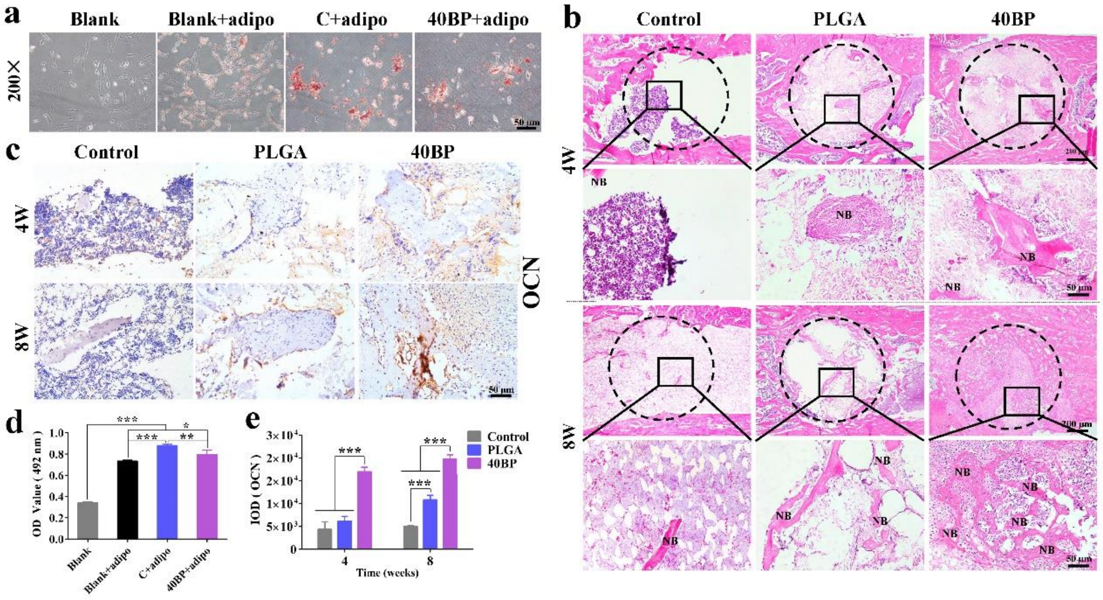

Supplementary Fig. 4. verification of SAON rat model cells by adipogenic induction staining and histological analysis of each group by H&E and immunohistochemical staining after 4 and 8 weeks. a. Adipogenic differentiation of WT rBMSCs (blank), WT rBMSCs with adipogenic induction medium (blank+adipo), rBMSCs from SAON rat model with adipogenic induction medium (C+adipo), rBMSCs from 40BP scaffold implanted to SAON rat model with adipogenic induction medium (40BP+adipo). **b.** H&E staining of bone-implant interface in different groups after 4 and 8 weeks. **c.** OCN immunochemistry staining of bone-implant interface in different groups after 4 and 8 weeks. **d.** Quantification analysis of lipid droplets OD 492nm in panel a. e. Quantification analysis of OCN stained area in panel c. n=3 independent samples, * p<0.05, ** p<0.01, *** p<0.001 by one-way ANOVA with Tukey's post hoc test.

Supplementary Table 1. The primer sequences used for real-time PCR

Gene	Forward primer	Reverse primer
M-IL-1β	5’-GCAACTGTTCCTGAACTCAACT-3’	5’-ATCTTTTGGGGTCCGTCAACT-3’
M-MCP-1	5’-TTAAAAACCTGGATCGGAACCAA-3’	5’-GCATTAGCTTCAGATTTACGGGT-3’
M-IL-6	5’-TAGTCCTTCCTACCCCAATTTCC-3’	5’-TTGGTCCTTAGCCACTCCTTC-3’
M-TNF-α	5’-GACGTGGAACTGGCAGAAGAG-3’	5’-TTGGTGGTTTGTGAGTGTGAG-3’
M-Arg-1	5’-CTGGCAGTTGGAAGCATCTCT-3’	5’-GTGAGCATCCACCCAAATGAC-3’
M-CD206	5’-CTCTGTTCAGCTATTGGACGC-3’	5’-TGGCACTCCCAAACATAATTTGA-3’
M-IL-10	5’-CTTACTGACTGGCATGAGGATCA-3’	5’-GCAGCTCTAGGAGCATGTGG-3’
M-TGF-β	5’-CCACCTGCAAGACCATCGAC-3’	5’-CTGGCGAGCCTTAGTTTGGAC-3’
M-GAPDH	5’-ACCCAGAAGACTGTGGATGG-3’	5’-TTCAGCTCAGGGATGACCTT-3’
H-PI3K	5’-CAGCGGACCTTTGAGGAGTT-3’	5’-AAATCACACTCGGCCACCTC-3’
H-IBSP	5’-CACTGGAGCCAATGCAGAAGA-3’	5’-TGGTGGGGTTGTAGGTTCAAA-3’
H-SPP1	5’-GAAGTTTCGCAGACCTGACAT-3’	5’-GTATGCACCATTCAACTCCTCG -3’
H-OPN	5’-CTCCATTGACTCGAACGACTC-3’	5’-CAGGTCTGCGAAACTTCTTAGAT-3’
H-Runx2	5’-CCGCACGACAACCGCACCAT-3’	5’-CGCTCCGGCCCACAAATCTC-3’
H-Osterix	5’-CACTTGAAGGAGGAGTTCGT-3’	5’-ATGAGACACCCGTCACTGGA-3’
H-ALP	5’-AGCACTCCCACTTCATCTGGAA-3’	5’-GAGACCCAATAGGTAGTCCACATTG-3’
H-BMP2	5’-ACTACCAGAAACGAGTGGGAA-3’	5’-GCATCTGTTCTCGGAAAACCT-3’
H-GAPDH	5’-AGAAAAACCTGCCAAATATGATGAC-3’	5’-TGGGTGTCGCTGTTGAAGTC-3’
